# Tissue-Engineered Vascular Graft of Small Diameter Based on Electrospun Polylactide Microfibers

**DOI:** 10.1155/2017/9034186

**Published:** 2017-11-08

**Authors:** P. V. Popryadukhin, G. I. Popov, G. Yu. Yukina, I. P. Dobrovolskaya, E. M. Ivan'kova, V. N. Vavilov, V. E. Yudin

**Affiliations:** ^1^Institute of Macromolecular Compounds, Russian Academy of Sciences, Bolshoy Pr. 31, Saint-Petersburg 199004, Russia; ^2^Peter the Great Saint-Petersburg State Polytechnical University, Polytechnicheskaya Str. 29, Saint-Petersburg 194064, Russia; ^3^Pavlov First Saint-Petersburg State Medical University, Leo Tolstoy Str. 6-8, Saint-Petersburg 197022, Russia

## Abstract

Tubular vascular grafts 1.1 mm in diameter based on poly(L-lactide) microfibers were obtained by electrospinning. X-ray diffraction and scanning electron microscopy data demonstrated that the samples treated at *T* = 70°C for 1 h in the fixed state on a cylindrical mandrel possessed dense fibrous structure; their degree of crystallinity was approximately 44%. Strength and deformation stability of these samples were higher than those of the native blood vessels; thus, it was possible to use them in tissue engineering as bioresorbable vascular grafts. The experiments on including implantation into rat abdominal aorta demonstrated that the obtained vascular grafts did not cause pathological reactions in the rats; in four weeks, inner side of the grafts became completely covered with endothelial cells, and fibroblasts grew throughout the wall. After exposure for 12 weeks, resorption of PLLA fibers started, and this process was completed in 64 weeks. Resorbed synthetic fibers were replaced by collagen and fibroblasts. At that time, the blood vessel was formed; its neointima and neoadventitia were close to those of the native vessel in structure and composition.

## 1. Introduction

In the modern vascular surgery, the problem of the development of vascular grafts with small diameter still exists. Low patency rates of the synthetic prostheses with diameter less than 5 mm are related, first of all, to development of neointimal hyperplasia at anastomosis sites and the absence of endothelial layer on the inner side of prostheses [1–4]. The problem of using autovenous material is its limited amount and high possibility of pathological changes in autovenous wall after implantation [5, 6]. As for pediatric vascular surgery, it is necessary to repeat reconstructive vascular operations due to the fact that nonresorptive synthetic prostheses cannot grow up and develop with a child organism [7]. Any attempts to create vascular grafts of small diameter by traditional methods were unsuccessful, since thromboses arose inside the grafts over a short period of time [8] because of low blood stream rate in these grafts. Currently, there are several techniques for developing artificial blood vessels and some of them are now undergoing clinical trials. The main methods are the following: obtaining tissue-engineered vascular grafts (TEVG) by layer-by-layer tissue engineering [9–14]; production of artificial vessels from granulation tissue [15–18]; use of decellularized transplants [19–21]; obtaining artificial vessels based on tubular bioresorbed polymer grafts [22–27].

The problem of thrombosis on early stages of implantation can be solved by using artificial vessels obtained by modern tissue engineering methods with the use of grafts made of biocompatible and bioresorbable polymer. Such artificial blood vessel should imitate structure and functions of the native vessel and be sensitive to neurohumoral action from recipient organism. The method consists in cultivating cells on bioresorbable graft in the bioreactor which imitates biological and mechanical factors providing proliferation and differentiation of the cells. It is expected that after implantation of this TEVG into recipient organism, biodegradation of polymer structures will be accompanied by the formation of a new vascular wall [28]. Another approach to development of artificial vessel includes implantation of a polymer graft into living organism where the cells from the surrounding tissues migrate to the graft and fill it forming a TEVG. Thus, functional tissues are formed and in parallel with this process; resorption of a polymer graft takes place under the action of active biological medium. When resorption is completed, a new blood vessel should be formed on the place of the graft. New artificial vessel should meet the following requirements: (1) to be biocompatible and infection-resistant; (2) to be hermetical and resistant to thrombosis (thus, the inner surface of the artificial vessel should be covered with endothelium); (3) to possess mechanical characteristics which allow carrying out surgical manipulations and also endure prolonged hydrodynamic loadings; (4) to possess vasoactive physiological properties (including ability to undergo spasm or dilatation as a response to nervous or chemical stimuli). Besides, it is necessary to have possibility of producing vascular grafts with various characteristics in sufficient amounts for solving any clinical problems [29]. Recently, there are a number of publications which describe attempts to omit the stage of cell cultivation on grafts* in vitro* and thus to simplify the technique and approximate it to clinical trials [30].

One of the promising methods to produce polymer vascular grafts is electrospinning. The method allows obtaining materials based on nano- and microfibers which demonstrate high porosity and specific surface area (the latter features are necessary for migration and proliferation of cells in graft volume) and simultaneously keep tightness with respect to blood [31–33]. The vascular grafts obtained by electrospinning possess the necessary mechanical characteristics. They are able to be integrated quickly into living organism, and their inner surface is covered with endothelium, which significantly reduces risk of thrombosis [34, 35].

The aim of the present work was development of a method for producing vascular grafts with small diameter based on poly(L-lactide) (PLLA) microfibers, studies of their structure, strength and deformation properties,* in vivo* investigation of biological tissue formation on the obtained grafts, and analysis of bioresorption mechanism.

## 2. Materials and Methods

### 2.1. Materials

The objects of the study were tubular grafts based on microfibers which were produced from PLLA «Purasorb PL-10» (Corbion Purac, Netherlands). Chloroform (Sigma-Aldrich, USA) was used as a solvent. All materials were of purissimus grade.

### 2.2. Processing of Nanofibers by Electrospinning

The microfibers were produced by electrospinning from the solution of PLLA in chloroform using a laboratory-scale Nanon-01A instrument (Japan). PLLA solution with a concentration of 15 wt.% was pumped through the die in the electrical field (*V* = 16 kV); the distance between the electrodes was 0.15 m; fiber deposition occurred on cylindrical electrodes. The rotational speed of cylindrical electrode (having a diameter of 1.1 mm) was 1500 rpm. Tubular samples with an inner diameter of 1.1 mm and wall thickness of 320 *μ*m were produced.

### 2.3. Investigation of Mechanical Properties

Mechanical properties of the grafts were studied using a universal Instron 5943 setup (United Kingdom) in the uniaxial tension mode at a rate of 10 mm/min. The Young modulus, tensile strength, and tensile deformation were determined for tubular samples based on PLLA with diameters of 1.1 mm and 850 *μ*m and wall thicknesses of 370, 320, and 250 *μ*m as well as for samples of native rat aorta. The length of test section of all tested samples was 20 mm.

### 2.4. Contact Angle Measurements

Wetting angle was measured on the surface of PLLA films and on the inner side of tubular samples from PLLA microfibers with the help of a DSA 30 instrument (KRUSS, Germany).

### 2.5. Investigation of Sample Structure

Structure of the tubular samples based on the PLLA microfibers was investigated using scanning electron microscope Supra 55 VP (Carl Zeiss, Germany). The sample surface was previously sputtered by platinum. Measuring of the wall thickness of tubular specimens, fiber diameter, and pore size (interfiber space) was performed using analysis of microphotographs.

Mean pore diameters and diameter of fibers were determined by image analysis (Scandium, ©OLYMPUS Soft Imaging Solutions) of SEM micrographs of tubular specimens. For this purpose SEM images of several areas perpendicular to the pore direction were captured. At least 20 pores were analyzed from different locations of the same sample.

X-ray diffraction (WAXD, Bruker D8 DISCOVER, Germany) was used for studying fine structure of the grafts.

Glass transition temperature, melting point, and crystallization temperature of PLLA were determined by differential scanning calorimetry (DSC) using a DSC 204 F1 Phoenix instrument (NETZSCH, Germany) in argon atmosphere.

### 2.6. Implantation Technique

Female rats (weight of 350 g) of a single genetic line were operated with inhalation anesthesia (1.5% isoflurane, induction of anesthesia, 3% isoflurane). After Y-shaped laparotomy, mobilization of an infrarenal aorta with ligation of lumbar arteries and the right renal artery clipping were made. The infrarenal aorta was replaced with the graft made of crystallized PLLA with an inner diameter of 1.1 mm and a length of 5 mm. An operating microscope, special tools, and an atraumatic needle (Prolene 9-0) were used. The mean diameter of the rat abdominal aorta was 1.0 mm. Anticoagulants were not applied. Surgical interventions were made in 42 female albino Wistar rats. Animals received free access to water and standard diet, which included PC-120-1 all-mash. The evaluations were made in 1, 4, 12, 24, 48, 56, and 64 weeks after operation. The conclusions were drawn from the experiments with 6 animals.

Ischemia time of the lower part of the body, the hind limbs, and the right kidney was about 50 minutes. In all trials, the hemorrhage through the anastomosis was not significant. Patency was assessed by classical technique immediately and 30 minutes after operation [36]. The abdominal wall was sutured layer by layer. The animals were kept in individual cages in a vivarium with free access to food and water. Postoperatively, the color and temperature of the skin of the hind limbs of the animals and their physical activity were examined.

All animals were treated appropriately according to Order number 1179 of Ministry of Health Care (10.10.1983); Order number 267 of Ministry of Health Care (19.06.2003); “Rules of Using Experimental Animals”; the principles of the European Convention (Strasbourg, 1986); and the Helsinki Declaration of the World Medical Association about humane treatment of animals (1996).

### 2.7. Angiographic Study

Angiographic studies were carried out with the aid of a Philips Allura Xper FD 20 setup (Netherlands) after open puncture of rats abdominal aorta in the area above prosthesis zone. The contrast substance Omnipaque (300 mg/mL) was used.

### 2.8. Microscopic Examination

In order to carry out histological examinations, the prosthesis and the surrounding portion of the native vessel were fixed in 10% neutral formalin phosphate buffer (pH 7.4) for 24 hours. Special histological technique with isopropyl alcohol or petroleum was used for embedding material into paraffin blocks. Paraffin sections having thickness of 5 *μ*m were cut and stained by hematoxylin and eosin. The Mallory and the Weigert-Van Gieson methods were used (Mallory trichrome and Weigert-Van Gieson, Bio-Optica, Italy) for visualization of connective tissue and elastic fibers.

To identify the endothelium, an immunohistochemical method was used and the immunoreactivity of the PECAM-1/CD31 protein was evaluated. After the standard dewaxing and rehydration procedures, the antigen was thermally smeared in pH 6.0 buffer (Diagnostic BioSystems, USA) for 60 min. The incubation was carried out for 30 minutes at room temperature. At the first stage, incubation of sections with polyclonal goat antibodies to PECAM-1/CD31 (Santa Cruz Biotechnology, USA) was carried out. Antibodies were diluted as 1 : 100 on a blocking solution (Diagnostic BioSystems, USA). To reveal the antigen-antibody complex, a set of reagents of Super Sensitive Polymer-HRP IHC Detection System (BioGenex, USA) was used. The preparations were additionally stained with Gill's hematoxylin (Bio-Optica, Italy).

Photographs were taken using a Leica DM750 optical microscope and an ICC50 camera (Leica, Germany). The tissue sections were observed under the microscope using the eyepiece N 10, and lenses N 4, 10, and 40. Morphometric analysis was performed using the “Videotest” system; eyepieces with magnification ×10 and lens N 40 were used.

## 3. Results and Discussion

According to the SEM data, the grafts obtained from PLLA solution by electrospinning consist of microfibers 1.5–4 *μ*m in diameter.

**Figure 1:**
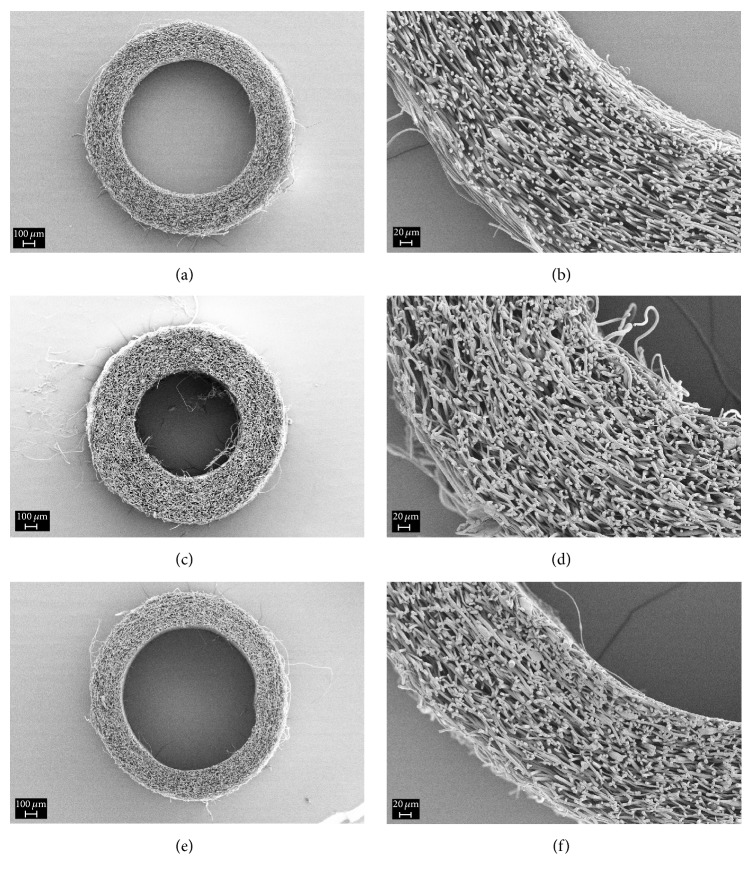
Cross sections of the grafts based on PLLA microfibers: the initial sample (a, b) and the sample crystallized in the free (c, d) and fixed (e, f) states.

Inner diameter of the grafts is 1.1 mm; wall thickness is 320 *μ*m. The microfibers have no preferential orientation and pores between them have average sizes of tens and hundreds of micrometers (Figures 1(a) and 1(b)). The X-ray structural analysis data demonstrated that the microfibers have amorphous structure.

After implantation of these samples into rats abdominal aorta, the prosthesis was deformed, and the wall integrity was affected during contact with sutures at anastomosis zones. Besides, a portion of fibers on the inner side of a tube was deformed during surgical manipulations; vascular lumen became smaller, and thus thromboses in early postoperative period were provoked. Implantation of these prostheses was difficult and inefficient.

It is well known that PLLA is a crystallizable polymer. It was revealed by DSC examination that PLLA is able to form crystalline phase inside the graft at temperatures close to its glass transition temperature (53–59°C). In this connection, there is a risk of spontaneous crystallization of the polymer at body temperature and under the action of biological media. Crystallization would be accompanied by shrinkage of microfibers and, correspondingly, by decrease in graft diameter; these processes could also lead to increasing risk of thromboses.

In order to improve mechanical properties and stability in biological medium, PLLA grafts were subjected to thermal treatment in free and fixed states at *T* = 70°C for 1 h. The DSC and X-ray diffraction data revealed that after thermal treatment at temperature above the glass transition point partial crystallization of the polymer occurred; degree of crystallinity of the treated PLLA samples was about 44%.

In the case of grafts treated in free state (as seen in Figures 1(c) and 1(d)), wall thickness increased from 320 *μ*m (for amorphous sample) to 370 *μ*m, and inner diameter decreased from 1.1 mm to 0.85 mm; the structure of graft wall became more loose and fibrous.

Thermal treatment of amorphous sample in the fixed state was carried out on a cylindrical mandrel 1.1 mm in diameter that led to decrease in graft wall thickness down to 250 *μ*m (Figures 1(e) and 1(f)) without decrease in the inner diameter; more dense fibrous structure was formed.

Mechanical characteristics of the thermally treated grafts were considerably better than those of the initial amorphous samples and exceeded the corresponding values for the native vessel (Table 1).

An important characteristic of biomaterials for tissue engineering is their wettability by water and other liquid media of an organism. On the one hand, high hydrophobicity of these materials prevents adhesion of cells on their surface and thus affects tissue formation. On the other hand, high hydrophilicity and, consequently, swelling and strong interaction between a graft and molecules of liquid environment can cause formation of thrombi [37]. The present studies demonstrated that the wetting contact angle of PLLA film after 1 h heat treatment was 80°. This value is typical of materials with rather low hydrophobicity. However, the contact angle of inner surface of the graft based on PLLA microfibers was 120°. Such value is characteristic of materials with high hydrophobicity and was reached due to surface relief. It is expected to reduce the risk of thrombosis during blood stream through the graft. However, it does not prevent further cell adhesion and endothelization of the surface graft. The most stable values of hydrophobicity were observed on samples crystallized in a fixed state. Besides, the increased initial mechanical characteristics for bioresorbable vascular grafts as compared to the native vessel are some advantage because the bioresorption process can go nonuniformly and not always qualitatively. Thus, a margin of safety is quite necessary. The best mechanical characteristics were obtained on samples crystallized in a fixed state (Table 1).

**Figure 2:**
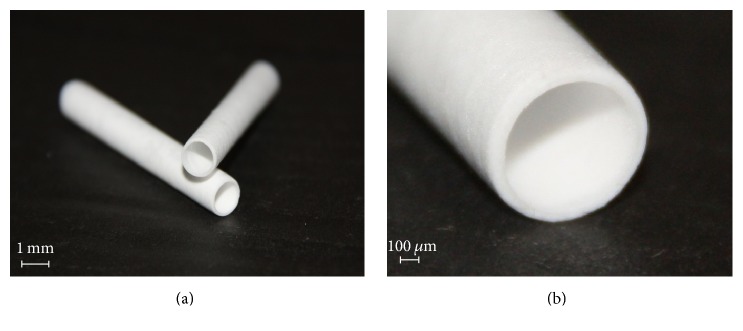
The grafts produced from the crystallized PLLA microfibers by electrospinning and photographed at low (a) and high (b) magnification.

On the basis of the above data of mechanical characteristics, hydrophobicity parameters, stability of internal diameter, and thickness and homogeneity of the wall, the grafts of 5 mm in length thermally treated in the fixed state were selected for in vivo experiments (Figure 2).

**Figure 3:**
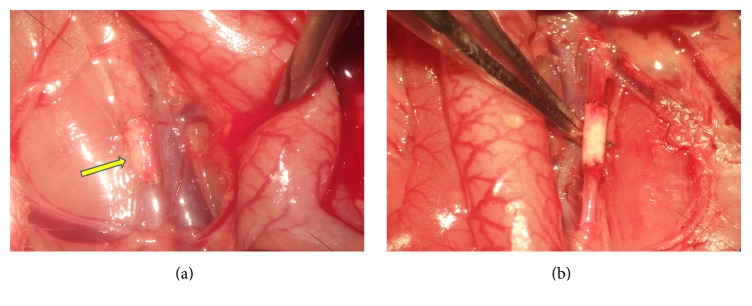
The graft four weeks after implantation covered with tissue (a) and separated (b). The arrow indicates the implanted graft.

The experiments involving implantation of the grafts into rat abdominal aorta demonstrated that in 1 week all grafts remained passable; aorta was adherent to the graft; removal of sutures did not cause destruction of anastomosis; no pathological influence on the surrounding tissue was revealed. Morphological analysis of the material showed that endothelial layer was formed on the inner side of the graft starting from distal and proximal anastomoses; the central part was covered with inhomogeneous network layer of fibrin. Between PLLA fibers, mainly from the adventitia side, fibroblast nuclei were found; thin collagen fibers started to appear. Giant polynuclear cells of foreign bodies were located on the outer side of the graft. The presence of these cells is typical of the organism's reaction to a foreign body, since they are actively involved in its destruction. However, in this case the cell sizes do not allow them to penetrate through the pores into the interior of the graft. In four weeks, all grafts remained passable; they were covered with connective tissue on the outside and adhered to connective tissue (Figure 3).

**Figure 4:**
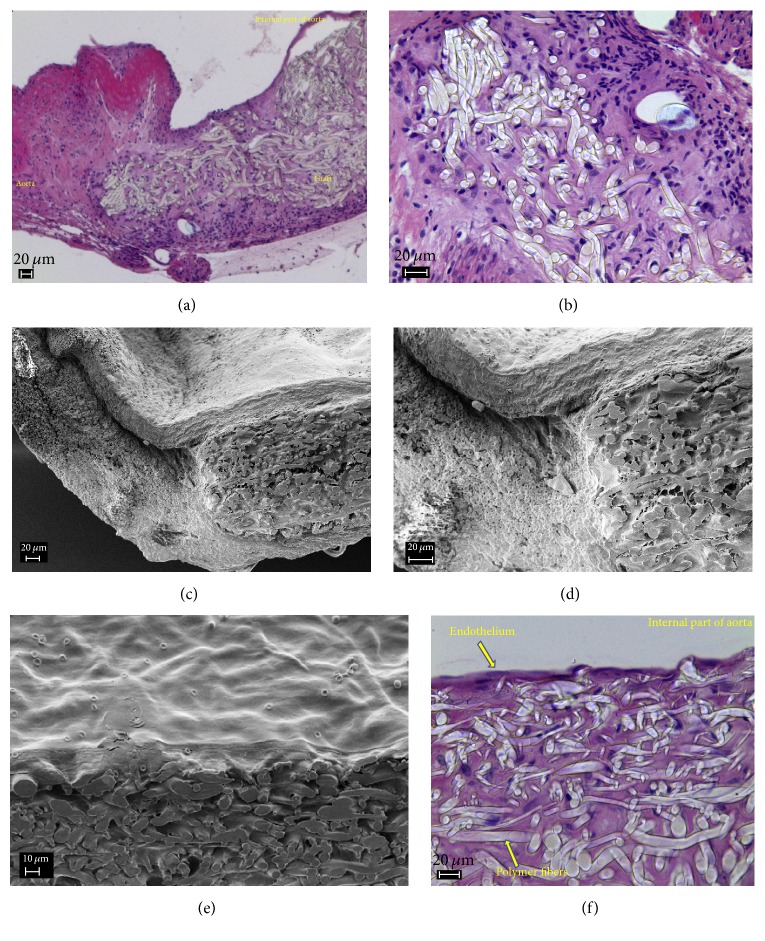
The results of histological and SEM studies of the vascular grafts after exposure for 4 weeks; anastomosis (a, b, c, d); fragment of the wall with neointima (e, f).

Histological studies showed that the graft was completely covered with endothelium. Subendothelial layer was formed along the whole length of the graft and contained thin network of the collagen fibers. The thickest neointima was formed in anastomosis zones (55 *μ*m), and its thickness gradually decreased towards the center of graft (down to 10 *μ*m). There were no signs of hyperplasia of the neointima. The whole width of the graft was filled with fibroblasts and pierced with thin collagen fibers. From the side of the adventitia, the collagen fibers were thicker, and the outer side of the graft was surrounded by a capsule formed from the connective tissue (Figure 4).

No immune response of an organism towards the graft was revealed. Bioresorption of the polymer was not observed either; structure of microfibers was retained.

Two of six grafts became thrombosed in 12 weeks. The grafts retained their shape and structure. The signs of beginning bioresorption were found; cross section of the fibers demonstrated low porosity.

Morphological studies revealed that endothelium and subendothelium containing collagen fibers covered the graft completely and neointima was formed. Within the thickness of the graft, between PLLA fibers, fibroblasts and the newly formed collagen fibers were located. Numerous giant polynuclear cells of foreign bodies can be seen from the adventitia side.

**Figure 5:**
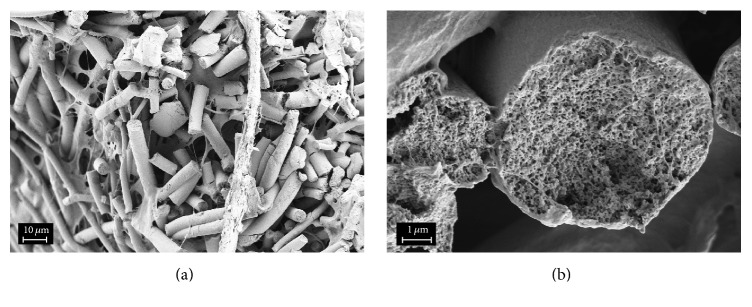
Fragment of the prosthesis wall in 24 weeks after implantation (a); fragment of the microfiber (b).

In 24 weeks after implantation, all grafts remained passable, numerous cross cracks were observed on microfibers, and a portion of fibers was fragmented (Figure 5(a)). Cross sections of the microfibers demonstrate pronounced porosity (Figure 5(b)) which was indicative of active bioresorption.

Histological analysis of the grafts performed after 24 weeks (similarly to the data obtained in the early postoperational period) showed completely formed endothelium and subendothelium consisting of loose collagen fibers. In one graft, completely formed plethoric blood vessels were revealed in the subendothelial layer. The grafts were completely occupied with fibroblasts and pierced with the collagen fibers. Over the whole volume of the grafts, numerous round cavities with diameter ranging from 1.750 to 7.320 *μ*m filled with weakly oxyphilic contents were observed between PLLA microfibers. These cavities were located chaotically and not surrounded by capsules; they destroy the structure of the wall of the new formed vessel that reduces its mechanical properties. Perhaps the appearance of cavities is associated with the resorption of polymer fibers [38]. In this regard, to exclude the risk of destruction of the vascular wall and the appearance of life-threatening bleeding, additional reliable strengthening of the vascular wall is necessary. On the outside of the grafts, the capsule of connective tissue containing giant polynuclear cells of foreign bodies was found.

In 48 weeks, one of six grafts was impassable and contained a thrombus which ran the entire length of the prosthesis. In passable grafts, microfibers were strongly fragmented and partially resorbed.

Morphological studies demonstrated that subendothelial layer was wide and consisted of densely packed collagen fibers and individual elastic fibers. In the subendothelial layer, calcium salts were deposited. Neomedia consisted of thick bundles of the collagen fibers which were randomly arranged. Fragments of PLLA fibers could be seen and mainly fibroblasts were located between them. Round cavities with weakly oxyphilic contents were observed over the whole graft volume; their area varied from 6.590 *μ*m^2^ to 11.500 *μ*m^2^. Adventitia consisted of loose fibrous connective tissue which contained vessels and giant polynuclear cells of foreign bodies.

In 56 weeks, all grafts of this group were still passable; in one sample, uniform hyperplasia of intima was revealed throughout the graft; its diameter decreased by 50%. Insignificant aneurisms were observed in 2 grafts.

**Figure 6:**
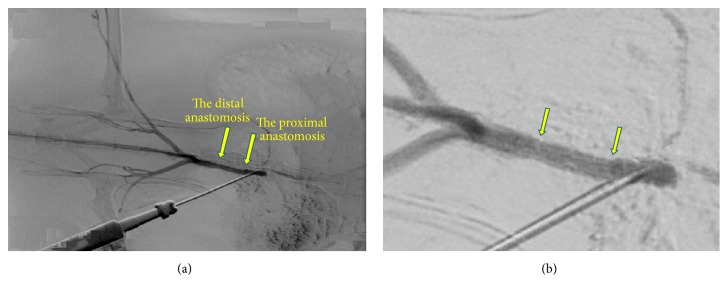
Intravital angiographic study performed in 56 weeks after implantation (a); the same sample shown at higher magnification (b). Arrows indicate the boundaries of the implanted graft.

According to the results of intravital angiographic study, the grafts were passable and conducted a pulse wave without difficulty; the prosthesis boundaries were barely distinguishable; there were no signs of stenosis or dilatation (Figure 6).

**Figure 7:**
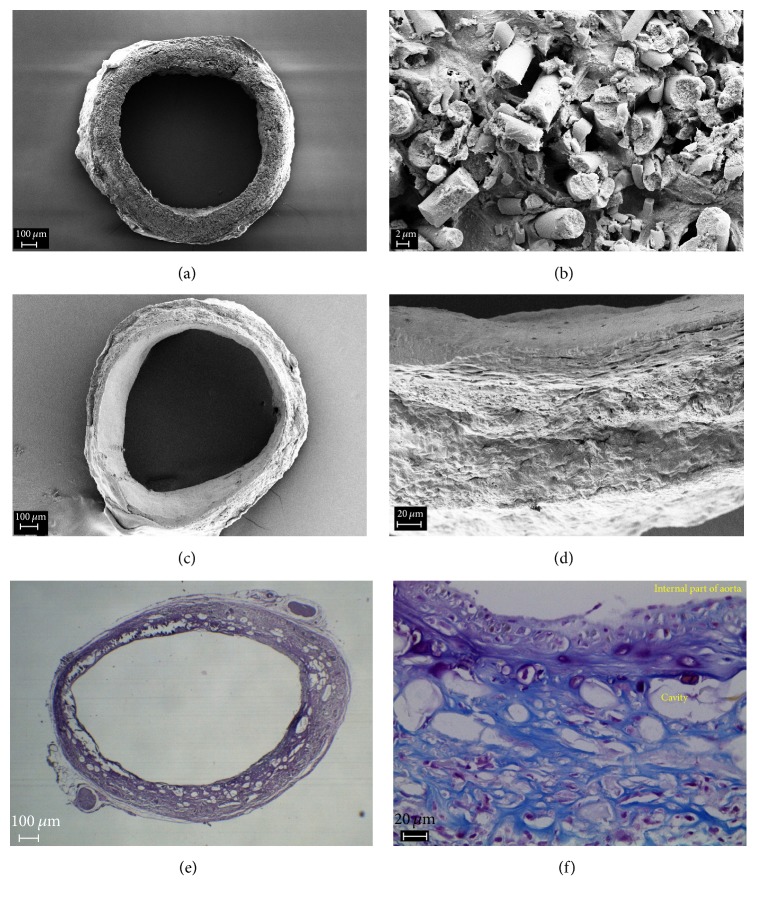
The graft in 56 weeks after implantation (a); fragment of the graft wall (b). The graft in 64 weeks after implantation (c, e); fragment of the graft wall (d, f). Histological sections (e, f) were stained according to the Mallory method.

Resorption of polylactide fibers was found to continue; longitudinal sizes of visible fragments of the microfibers varied from 1 to 10 *μ*m (Figures 7(a) and 7(b)).

Histological sections of the studied samples were similar to those obtained earlier. Endothelium and subendothelium consisting of collagen and the elastic fibers were revealed throughout the graft. However, neointima thickness was not the same in various parts. Neomedia consisted of disordered bundles of the collagen fibers, fibroblasts, and fragments of the PLLA fibers. The area of newly formed cavities between the microfibers ranged from 3.360 *μ*m^2^ to 7.040 *μ*m^2^. Vessels and giant polynuclear cells of foreign bodies were revealed in adventitia consisting of the connective tissue along the whole surface of the graft.

**Figure 8:**
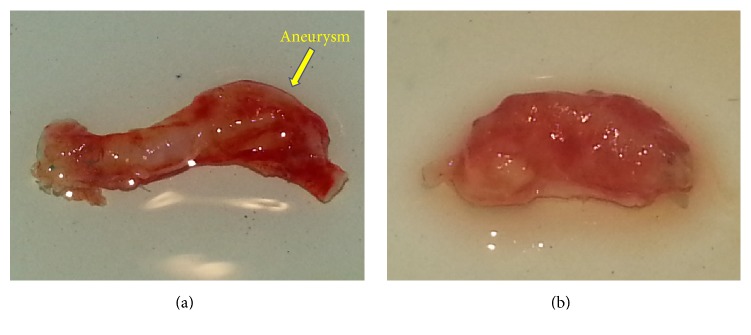
The graft in 64 weeks after implantation; aneurysms of various degrees: minimal (a) and maximal (b).

In 64 weeks, all grafts remained passable and consisted completely of newly formed tissues with the wall width varying from 100 to 160 *μ*m (Figures 7(c) and 7(d)). All newly formed vessels demonstrated aneurysms of various degrees (Figure 8). The presence of an aneurysm creates a risk of thrombosis, thromboembolism, and rupture of an aneurysm. These complications can threaten the patient's life and require urgent treatment [38, 39].

Neointima consisted of the endothelium and subendothelial layer which included collagen and elastic fibers. Neomedia consisted mainly of the fibroblasts. Collagen fibers formed disordered bundles. No PLLA fiber fragments were revealed. The area of cavities in the newly formed neomedia reached the values from 2.690 *μ*m^2^ to 21.700 *μ*m^2^ (Figures 7(e) and 7(f)). Neoadventitia consisted of loose fibrous connective tissue with vessels. Giant polynuclear cells of foreign bodies were observed in neoadventitia.

## 4. Conclusions

The studies of the vascular grafts consisting of PLLA microfibers by electrospinning technique demonstrated that the most promising mechanical and performance characteristics were inherent in the grafts with partially crystalline microfibers; this structure was obtained after thermal treatment of the initial tubular sample in the fixed state.

The grafts implanted into the rat's abdominal aorta did not cause pathological reactions in the rats; in 4 weeks, their inner side became completely covered with endothelium, and the whole wall was occupied by fibroblasts. These facts indicated good integration between the graft and the body of the rats. The presence of the endothelium layer in early postoperational period facilitated high stability of the graft against thrombosis.

First signs of bioresorption of microfibers were observed in 12 weeks after implantation; bioresorption continued until the 56th week and did not cause visible pathological reactions. Resorbed fibers were replaced by collagen fibers and fibroblasts. It is important to note that resorption rate of polymer graft did not exceed the rate of growth of cells and tissues inside it.

The blood vessels had been formed in 64 weeks after implantation; these vessels had intima and adventitia almost similar to those of the native vessel. It should be noted that neomedia consisted of disordered bundles of collagen fibers, fibroblasts, and oxyphilic cavities. The formation of this partially completed structure led to reduction of mechanical characteristics of vessels and appearance of aneurysms; at the same time, very high total permeability of the grafts was observed (93%).

One can conclude from the obtained results that PLLA grafts produced by electrospinning are promising for clinical uses, although some methods for strengthening walls of the newly formed blood vessels should be developed.

## Figures and Tables

**Table 1:** Mechanical characteristics of the grafts based on PLLA microfibers.

Medium	Wall thickness, *μ*m	Tensile strength, МPа	Young's modulus, МPа	Elongation, %
Native rat aorta	150	2.27 ± 0.56	17.3 ± 5.1	139 ± 32
The initial PLLA graft	320	2.64 ± 0.29	27.0 ± 2.8	258 ± 24
PLLA graft thermally treated in the fixed state	250	3.63 ± 0.41	145 ± 11	175 ± 18
PLLA graft thermally treated in the free state	370	2.61 ± 0.36	109 ± 13	96.3 ± 21
